# ABO phenotypes and malaria related outcomes in mothers and babies in The Gambia: a role for histo-blood groups in placental malaria?

**DOI:** 10.1186/1475-2875-5-72

**Published:** 2006-08-17

**Authors:** María-Paz Loscertales, Bernard J Brabin

**Affiliations:** 1 Child and Reproductive Health Group, Liverpool School of Tropical Medicine, Liverpool, UK; 2 Emma Kinderziekenhuis, Academia Medical Centre, University of Ámsterdam, The Netherlands; 3 Department of Community Child Health, Royal Liverpool Children's Hospital, Alder Hey NHS Trust, Liverpool, UK

## Abstract

**Background:**

Host susceptibility to P.*falciparum *is critical for understanding malaria in pregnancy, its consequences for the mother and baby, and for improving malaria control in pregnant women. Yet host genetic factors which could influence placental malaria risk are little studied and there are no reports of the role of blood group polymorphisms on pregnancy outcomes in malaria endemic areas.

This study analyses the association between ABO blood group phenotypes in relation to placental malaria pathology.

**Methods:**

A total of 198 mother/child pairs delivering in Banjul and the Kombo-St Mary District (The Gambia) were analysed. ABO blood group was measured by agglutination. Placental malaria parasites wee enumerated and the presence of malaria pigment noted. Birth anthropometry was recorded and placental weight. Maternal and infant haemoglobin was measured.

**Results:**

89 (45%) subjects were primiparae and 110 (55%)multiparae. The ABO phenotype distribution was 38(A), 52(B), 6(AB) and 102(O). Placental histo-pathology showed active placental malaria in 74 (37%), past infection in 42 (21%) and no infection in 82 cases (41%). In primiparae blood group O was associated with a higher risk of active infection (OR = 2.99; 95% CI = 1.24–7.25), and a lower risk of past infection (OR = 0.31, 0.10–1.01, p < 0.05). In multiparae the O phenotype was associated with reduced prevalence of active or past placental infection (OR = 0.45; 95% CI 0.21–0.98). The mean feto-placental weight ratio was significantly higher in multiparae with group O women compared to non-O phenotypes (5.74 vs 5.36; p = 0.04). Among primiparae with active placental infection, mean birth weight was higher in children of mothers with the O phenotype (p = 0.04).

**Conclusion:**

These results indicate that blood group O was significantly associated with increased placental malaria infection in primiparae and reduced risk of infection in multiparae. This parity related susceptibility was not present with other ABO phenotypes. Cell surface glycans, such as ABO and related antigens have special relevance in reproductive biology and could modulate specific cell interactions as those associated with the pathogenesis of placental malaria.

## Background

In malaria endemic areas, the increased risk of *P. falciparum *infection during pregnancy, which is associated with placental parasitaemia, imposes a heavy burden on the health of mothers and newborns [1,2], contributing to maternal anaemia, low birth weight, and infant mortality especially among primiparae [3,4]. At present, available preventive and therapeutic tools can only achieve a partial reduction in the health hazards caused by placental malaria [5,6]. In this context, a better knowledge of host susceptibility to placental *P*.*falciparum *infection is central for improving understanding of malaria in pregnancy, as a basis for improved control.

There is increasing evidence that both the risk of acquiring *P. falciparum *infection, and the risk of developing severe complications are determined by host genetic factors [7]. The protective role of several erythrocytic variants, some of them related to blood groups, is one of the best examples of this genetic modulation [8]. These include haemoglobins S, C and E, α and β thalassaemias, Glucose-6-phosphate dehydrogenase deficiency, Southern Asian Ovalocytosis, and Glycophorins A, B and C variants, all of which influence malaria pathogenesis [9].

ABO blood groups are carbohydrate histo-blood antigens that are also expressed in many tissues and which have important roles in modulating protein activities both in infection and in some types of cancer [10]. These antigens are formed by terminal glycosylation of glycoproteins and glycolypid chains present on cell surfaces. Glycosylation modulates all kinds of cell-to-cell interactions and this may be relevant in malaria pathophysiology, where adhesion has been increasingly implicated in disease severity. Studies examining the effects of the ABO blood group phenotype on malaria risk in non-pregnant subjects, have shown inconsistent results [11-13]. Blood group A has been reported as a risk factor for severe malaria [14], and as a co-receptor for *P*.*falciparum *resetting [15], whereas blood group O may offer some protection against severity of disease [16].

No studies have been identified in the literature assessing associations of blood group types with placental malaria, despite the fact that placental parasites are unable to rosette [17], in contrast to isolates from non-pregnant subjects. Cell surface glycans have an essential role in reproductive biology, and the adhesion and implantation of the blastocyst is partly mediated by carbohydrates with blood group specificity [18]. Each mammalian species has its own glycotype at the feto-maternal interface, and this variation depends on both evolution and the environment [19]. ABO histo-blood groups and related antigens are expressed in the endometrium and are modulated by the hormonal environment [20], but are not expressed in the placenta and fetal endothelium where only other related blood groups can be detected in the interstitial trophoblast directly apposed to the maternal decidua [21]. In contrast, examination of the glycan expression at the feto-maternal interface using lectins, some with ABO determinant specificity have shown binding with placental structures [22,23].

In this analysis we present the first report to describe the association between ABO phenotypes, placental malaria and pregnancy outcomes.

## Materials and methods

### Study sample

Data from a group of 198 mother/child pairs were analysed from a cross-sectional study of placental malaria undertaken in the Gambia. The original descriptive study assessed serum Ig levels in mothers and newborns in relation to placental parasitaemia. The primary outcomes and a detailed description of the methodology are outlined in the original publication [24].

Mothers and their newborn babies were included in the study from September 1967 to May 1968 in Banjul and the Kombo-St Mary District (The Gambia). In this area malaria is endemic with peak transmission during and shortly after the rainy season which lasts from July to October [25]. Mothers and babies were examined soon after delivery at the Royal Victoria Hospital, Banjul, or in one of the maternal and child welfare centres in the Kombo-St Mary District. Basic demographic information was recorded. The weight (g) and length (cm) of the baby were measured by a physician within a few hours of birth. Placentas were transported twice daily to the Medical Research Council Laboratories where they were weighed and examined for placental pathology. Placental blood and maternal and neonatal peripheral venous blood were collected, and blood films were prepared and stained by standard methods. Parasitaemia was counted in the peripheral blood against the number of white cells, and in the placental blood by assuming that 1 parasite per 100 oil-immersion fields represented a density of 10 parasites/mm^3 ^blood. Maternal and infant haemoglobin was measured.

Data were entered on punch cards and for all births the following data was available: maternal age, parity, haemoglobin and peripherical parasitaemia; the babies gender, weight, length and haemoglobin. The ABO and Rhesus blood group phenotype of mother and baby were recorded and the placental weight (g), parasite count and presence of malaria pigment. All data was entered into a SPSS data file.

### Clinical definitions

Placental malaria definition was based on the pathological classification of Bulmer et al [26], which comprises the following groups: non-infected; acute infection (presence of parasites without pigment); chronic infection (presence of parasites and pigment); past infection (parasites not present; pigment present). Acute and chronic placental malaria were also grouped as 'active' infection.

### Statistical analysis

Dichotomous variables were assessed with chi-square or Fisher exact tests, with p values less than 0.05 considered statistically significant. Odds ratios and 95% confidence intervals were estimated. Differences between means were assessed by ANOVA where data was normally distributed, or the Mann-Whitney/Wilcoxon Test. Multiple linear regression was used to analyse factors associated with anthropometric outcomes. Factors included were those significant at p = 0.1 in the univariate analysis. For anthropometric variables the factors included where: maternal blood group (O versus non O), parity, placental infection, maternal haemoglobin, maternal peripheral parasitaemia and maternal height.

## Results

Eighty-eight of the 198 women were primiparae (45%) who had a mean age of 17.8 years (SD ± 1.8). The mean age of multiparae was 29.7 years (± 6.1). Placental malaria was present in 116 cases (59%), with active placental malaria infection in 74 (37.4%), of which 26 (13.1%) were acute and 48 (24.2%) chronic infections. Past infection was detected in 42 women (21.2%). In the remaining 82 cases (41%) there was no evidence for current or previous placental malaria. Among primiparae, 56 (63%) had evidence of placental infection of which 41 (47%) were active, 12 (14%) acute and 29 (33%) chronic infections. Among multiparae 60 (55%) had evidence of placental infection with 33 (30%) active, 14 (13%) acute and 19 (17%) chronic (Table 1). Primiparae were at increased risk for active placental malaria (OR 1.99, 95% CI 1.11 – 3.57), and more specifically the chronic infection (OR 2.32, 95% CI 1.19–4.5).

**Table 1:** ABO phenotype by placental malaria category and parity group

Parity	Placental malaria		Phenotype N (%)				OR 95% CI O/non O
		
	Type	No	A	B	AB	O	
**Primiparae**	Active	41	6 (37)	6 (30)	0 (0)	29 (58)	2.99, 1.24–7.25
	Past	15	4 (25)	4 (20)	2 (100)	5 (10)	0.3, 0.1–1.0
	None	33	6 (37)	10 (50)	0 (0)	16 (32)	0.65, 0.27–1.55
	**Total**	**88**	16	20	2	50	
							
**Multiparae**	Active	33	6 (27)	11 (29)	2 (50)	14 (27)	0.75, 0.33–1.7
	Past	27	8 (36)	10 (31)	0 (0)	9 (17)	0.47, 0.19–1.15
	None	50	8 (36)	11 (34)	2 (50)	29 (56)	2.22, 1.03–4.78
	**Total**	**110**	22	32	4	52	

Brackets: percentage

The male/female ratio was significantly increased in active placental malaria OR = 1.87 (95%CI 1.04–3.39), p = 0.04 and decreased in past infection OR = 0.45 (95%CI 0.22–0.90), p = 0.02.

There was no significant association between O or non-O blood groups and placental infection when parity was not considered, except for past placental malaria which was less prevalent in women with blood group O (OR 0.39, 95% CI 0.18–0.84, p < 0.01). In primiparae blood group O was associated with more active placental malaria (OR 2.99, 95% CI 1.24–7.25, p < 0.05) and less past placental malaria (OR = 0.31, 0.10–1.01, p < 0.05) than in non-O women. In multiparae, blood group O was significantly associated with non-infected placentae (OR 2.22, 95% CI 1.03–4.78, p < 0.05) compared to non-O women (Table 1). The effect of parity on the risk of placental infection was significant for the O blood group sub-population with lower risk of active infection in multigravidae (OR 0.27, 95% CI 0.12–0.62, p < 0.01) compared with primiparae of the same blood group. For non-O blood groups, there were no significant parity differences in risk of active (OR 1.06, 95%CI 0.44–2.59, p = 0.9) or past placental infection (OR 0.79, 95%CI 0.32–1.98, p = 0.6). The effect of parity on the risk of active placental infection was significantly different for the two sub-populations O and non-O, (Chi-square 4.98, p = 0.03).

Table 2 summarises birth outcomes by blood group phenotype. In primiparae, with active placental infection, mean birth weight was significantly higher in babies born to blood group O mothers (2893 g versus 2639 g, p = 0.04).

**Table 2:** Mean birth outcomes by parity and maternal blood group phenotype

				**Malaria Parameters : MEAN (SD)**							
				
**Parity**	**Placental Malaria type**	**Blood group**	**n**	**Maternal Hb g/dl**	**Infant Hb g/dl**	**Birth weight g**	**Length cm**	**Placental weight g**	**Ponderal index (‡)**	**Feto-placental weight ratio**	**Parasite count (log 10)**
**Primiparae**	**Active (†)**	**O group**	**29**	11.65 (1.6)	18.5 (3.1)	2893 (362)*	49.0 (1.8)	559 (123)	2.51 (0.24)	5.26 (0.9)	2.74 (1.36)
		**non-O**	**12**	10.53 (3.1)	19.4 (3.0)	2639 (323)*	47.9 (1.9)	513 (115)	2.45 (0.15)	5.3 (0.9)	3.18 (1.48)
	
	**Past or no infection**	**O group**	**21**	12.0 (1.3)	18.2 (2.2)	2941 (455)	48.6 (1.6)	544 (135)	2.59 (0.22)	5.62 (1.22)	
		**non-O**	**26**	11.8 (2.3)	18.2 (2.0)	2894 (430)	48.9 (1.8)	559 (100)	2.5 (0.21)	5.28 (0.9)	

**Multiparae**	**Active**	**O group**	**14**	11.9 (1.4)	18.5 (2.0)	3151 (705)	49.7 (2.4)	578 (124)	2.55 (0.26)	5.53 (1.15)	2.28 (1.19)
		**non-O**	**19**	12.3 (1.5)	18.7 (2.5)	3366 (312)	50.0 (1.8)	652 (169)	2.68 (0.20)	5.39 (1.1)	2.53 (1.18)
	
	**Past or no infection**	**O group**	**38**	12.2 (1.5)	18.6 (2.1)	3315 (537)	49.8 (2.3)	580 (121)	2.67 (0.24)	**5.82 (0.85)§**	
		**non-O**	**39**	11.8 (1.5)	18.7 (2.3)	3093 (521)	49.1 (2.0)	588 (115)	2.67 (0.23)	**5.34 (0.89)§**	

†Active: acute and chronic infection

‡Ponderal Index = weight/length3

* p value = 0.04

§p value = 0.02

Maternal haemoglobin (Hb) concentration was higher in O type mothers with active placental malaria (11.65 g/dl, versus 10.53 g/dl) although this difference was not statistically significant (p = 0.48). There were no significant differences in mean infant haemoglobin, infant length, placental weight, ponderal index or placental parasitaemia prevalence by blood group category (table 2).

**Figure 1:**
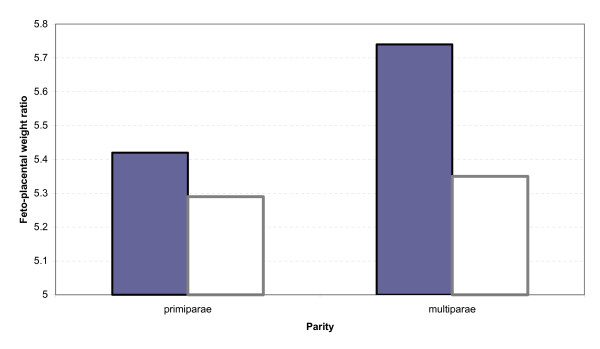
Mean feto-placental weight ratio in primiparae and multiparae according to blood group. Shaded blood group O, white non-O groups

Mean feto-placental weight ratio was increased in blood group O compared to non-O (p = 0.06) and was significant in multiparae (5.74 versus 5.36; p = 0.04), (Figure 1). Blood group phenotype (O versus non-O) was an independent predictor of the higher feto-placental weight ratio, (linear regression coefficient = 0.26, p = 0.04). The other malaria parameters, did not show any significant associations with blood groups on multivariate analysis.

The Rhesus blood group type showed no significant associations in relation to placental malaria categories or birth outcomes.

## Discussion

Blood group O was associated with an increased prevalence of active placental infection in primiparae and with a reduced risk of placental malaria in multiparae. Placental parasitaemia occurs at least twice as frequently in primiparae but only among blood group O women. This effect of parity, that is one of the cardinal features of placental malaria, was not observed in non-O phenotypes for any of the placental malaria histological types.

In multiparae and specifically in mothers with past or non-infected placentae blood group O was also associated with higher mean feto-placental weight ratios compared to non-O individuals. These differences associated with multiparity can be interpreted to represent a parity-specific association of the blood group O phenotype with the protective malarial immunity which occurs in multigravidae [3]. Mean birth weight was also significantly increased in babies of primiparae with the O phenotype, suggesting that despite the greater susceptibility of primigravidae to placental malaria, the O phenotype may be partially protective leading to improved birth anthropometry.

In non-pregnant individuals blood group O has been associated with reduced risk of severe clinical malaria compared to patients with groups A or B [14,16], but this occurred without clear evidence for reduced prevalence of parasitaemia associated with blood group O [12,13]. This would suggest that the ABO phenotypes may be associated more with modification of disease severity than with infection risk. None of these women had HIV infection as the study was conducted in the early 1970s before this human immunodeficiency virus was described or transmitted. This indicates that these results are not confounded by an immunosuppressive effect of HIV virus infection.

This is the first time an association of birth outcomes with placental malaria and maternal ABO phenotype has been reported. This raises the question of the possible mechanisms underlying the association. A central mechanism in the pathogenesis of placental malaria relates to the cytoadherence of infected red cells to the syncytiotrophoblast which is in contact with maternal blood. The parity related susceptibility to placental malaria is partly dependent on the selection of *P*.*falciparum *isolates which bind to trophoblast via chondroitin sulphate A (CSA) [27] and hyaluronic acid [28]. Infected red cells can also bind to the proteoglycan thrombomodulin, present on endothelial cells and placental syncytiotrophoblast, via CSA side chains [29]. Soluble adhesion molecules and endothelial markers (including won Willebrand factor and E-selectin) are associated with ABO phenotypes, especially thrombomodulin which is lower in group O and A than B plasma (P < 0.001) [30].

Duffy binding-like domains (DBL) of the *P*. *falciparum *erythrocyte membrane protein 1 (PfEMP1) mediate binding to several independent host receptors [31] and in placental isolates it is the DBL-γ3 domain which binds to chondroitin sulfate A (CSA) in syncytiotrophoblast [32,33]. However, there is no published evidence of an association between DBL-γ3 and the ABO phenotype, although it has been shown that the DBL1α domain has an affinity for the blood group A antigen [34].

The interaction between the ABO histo-blood group and placental malaria could relate to generic mechanisms affecting *P*.*falciparum *infection. For example the differential affinity of each phenotype for *A*.*gambiae *[35], or antigen sharing between ABO phenotypes and *P*.*falciparum *leading to changes in immune response [36], or an association with Glycophorin A (GPA) which is an important determinant for *P*.*falciparum *entry into the red cell through GPA sialic acid, as ABH antigens have been described in the O-glycans of glycophorin A [37]. In addition ABO phenotypes differ in sialic acid content and composition, with group O showing the highest membrane content, but a lower percentage of sialoglycoproteins [38].

It is well known that host genetic factors modulate the risk and severity of infection. In most cases these are genetic variants with subtle effects on the regulation or function of specific mediators which are often difficult to demonstrate in epidemiological studies [39]. As there is increasing evidence that cell adhesion plays a decisive role in placental malaria pathophysiology, it is clear that cell surface glycans, such as the ABO and related antigens which have special relevance in reproductive biology, could modulate some of those specific cell interactions. Their association with placental malaria risk, birth outcomes and parity related susceptibility, provides a new insight into these interactions and into the role of glycosylation and host-specific genetic factors in placental malaria.
